# Dr. John I. Wheeler

**Published:** 1909-01

**Authors:** 


### OBITUARY

Dr. John I. Wheeler died at his home, Chatham, N. Y., December 3, 1908, in the prime of his usefulness at the age of 58, after an abrupt and brief illness from pneumonia. He was at the time of his death, Director of the Division of Communicable Diseases of the Department of Health; a vice-president of the State Medical Society; a member of various professional and civic organisations. For nearly thirty years he had been a Trustee of Public Schools, and the admirable buildings and system of school and library of his village are a monument most prized by him of devoted work in this direction. He was a good physician with wide practice in the country about him; he was much sought as an adviser and leader in professional work by his associates; he had technical capacity and wise judgment, with a pleasing manner and address, and with a good heart for everyone. He was a rare man who had done good work in a good hearted way and his death leaves a large gap. The day of his death, all work ceased in the village and there was a great gathering from far and near to pay him a last tribute.

